# A prognostic pyroptosis-related LncRNA classifier associated with the immune landscape and therapy efficacy in glioma

**DOI:** 10.3389/fgene.2022.1026192

**Published:** 2022-10-24

**Authors:** Jiasheng Zhong, Jie Liu, Zhilin Huang, Yaofeng Zheng, Jiawen Chen, Jingsen Ji, Taoliang Chen, Yiquan Ke

**Affiliations:** The National Key Clinical Specialty, The Engineering Technology Research Center of Education Ministry of China, Guangdong Provincial Key Laboratory on Brain Function Repair and Regeneration, Guangzhou, China

**Keywords:** long nonconding RNA(lncRNA), pyroptosis, glioma, prognostic classifier, tumor immune microenvironment

## Abstract

**Background:** Glioma has the highest fatality rate among intracranial tumours. Besides, the heterogeneity of gliomas leads to different therapeutic effects even with the same treatment. Developing a new signature for glioma to achieve the concept of “personalised medicine” remains a significant challenge.

**Method:** The Cancer Genome Atlas (TCGA) and the Chinese Glioma Genome Atlas (CGGA) were searched to acquire information on glioma patients. Initially, correlation and univariate Cox regression analyses were performed to screen for prognostic pyroptosis-related long noncoding RNAs (PRLs). Secondly, 11 PRLs were selected to construct the classifier using certain algorithms. The efficacy of the classifier was then detected by the “timeROC” package for both the training and validation datasets. CIBERSORT and ESTIMATE packages were applied for comparing the differences (variations) in the immune landscape between the high- and low-risk groups. Finally, the therapeutic efficacy of the chemotherapy, radiotherapy, and immunotherapy were assessed using the “oncoPredict” package, survival analysis, and the tumour immune dysfunction and exclusion (TIDE) score, respectively.

**Results:** A classifier comprising 11 PRLs was constructed. The PRL classifier exhibits a more robust prediction capacity for the survival outcomes in patients with gliomas than the clinical characteristics irrespective of the dataset (training or validation dataset). Moreover, it was found that the tumour landscape between the low- and high-risk groups was significantly different. A high-risk score was linked to a more immunosuppressive tumour microenvironment. According to the outcome prediction and analysis of the chemotherapy, patients with different scores showed different responses to various chemotherapeutic drugs and immunotherapy. Meanwhile, the patient with glioma of WHO grade Ⅳ or aged >50 years in the high risk group had better survival following radiotherapy.

**Conclusion:** We constructed a PRL classifier to roughly predict the outcome of patients with gliomas. Furthermore, the PRL classifier was linked to the immune landscape of glioma and may guide clinical treatments.

## Introduction

Glioma represents the highest morbidity, incidence, and fatality rates compared to other intracranial tumours, with an annual incidence of 3–6.4/100,000 persons, accounting for 23.3% of brain tumours and 78.3% of malignant tumours (Sung et al., 2021). Moreover, gliomas are highly malignant and invasive, making them the leading cause of death associated with intracranial malignant tumours (Ostrom et al., 2019). The prognosis of low-grade glioma (LGG) is relatively good. The median overall survival (OS) of WHO grade 2 glioma is around 11 years, whereas that of WHO grade 3 glioma is 3 years (Smoll et al., 2012). The median OS of glioblastoma (GBM) is just 19.2 months (Tesileanu et al., 2020). Based on pathologic characteristics and molecular alterations, the 2021 World Health Organisation (WHO) classification of gliomas brings meaningful instructions to clinical practice (Louis et al., 2021). However, the prognostic classifier based on accurate tumour-specific biomarkers of glioma for personalised precision is still poor. Therefore, the construction of novel prognostic signatures and the direction of clinical treatment remain priorities.

Long noncoding RNAs (lncRNAs), a kind of RNA void of protein-coding function, are involved in tumour progression mechanisms, including proliferation, apoptosis, invasion, and migration (Gou et al., 2018). In gliomas, the involvement of lncRNA is not fully grasped. Research illustrates that lncRNA PCED1B-AS1 can up-regulate hypoxia-inducible factor 1-alpha (HIF-1α) expression, thus promoting cell proliferation, glucose uptake, and lactic acid release in glioma cells (Yao et al., 2020). Meanwhile, some lncRNAs also function as biological markers for predicting glioma patients’ prognoses. For example, the expression of insulin-like growth factor binding protein 7-antisense 1 (IGFBP7-AS1) is a biomarker correlated with a dismal prognosis for glioma individuals (Li et al., 2019). Furthermore, mounting data show that lncRNAs participate in pyroptosis regulation. Metastasis-associated lung adenocarcinoma transcript 1 (MALAT1) is capable of activating the nucleotide oligomerisation domain-like receptor family pyrin domain containing 3 (NLRP3) inflammasome by adsorbing microRNA (miRNA)-133 as a miRNA sponge through the competing endogenous RNA mechanism during cardiac ischaemia and reperfusion injury (Yu et al., 2018). XLOC_000647 has been proven to decrease the proliferative and invasive capacity of pancreatic cancer cells by reducing NLRP3 expression (Hu et al., 2018).

**FIGURE 1:**
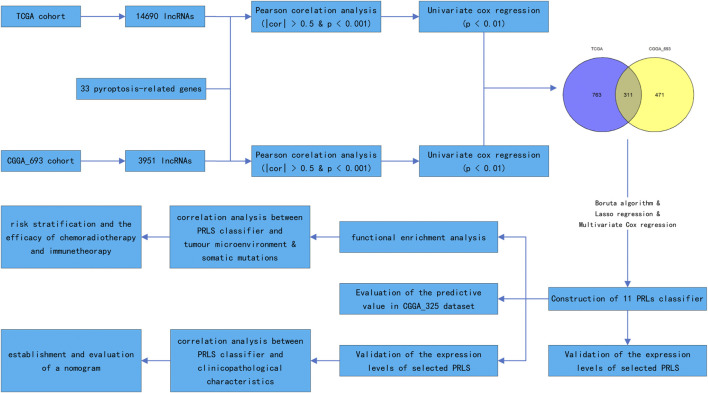
Research procedure shown as a flowchart.

Pyroptosis is not the same as the other types of cell death, including necrosis, apoptosis, and necrotic apoptosis, in that it releases inflammatory mediators regulated by gasdermin (Shi et al., 2017). The occurrence of the pyroptosis is the first to activate caspase 1 and caspase 4/5/11 *via* the classical and non-classical pathways to cleave gasdermin D (GSDMD) (Cheng et al., 2017; Karki and Kanneganti, 2019). After cleaving GSDMD, the N-terminal segment of GSDMD is implicated in the formation of oligomers and the binding of these oligomers to the cell membrane. This results in the generation of pores in the cell membrane and the production of inflammatory mediators, such as interleukin (IL)-1β and IL-18. Another mechanism for pyroptosis activation is the stimulation of caspase 3-induced cleavage of gasdermin E to produce its N-terminal products, resulting in cell perforation (Wang et al., 2017). The strong and complicated relationship between pyroptosis and tumourigenesis and tumour development has not been elucidated (Xia et al., 2019). Inflammatory mediators released from cells that undergo pyroptosis might promote tumourigenesis and induce drug resistance. On the other hand, pyroptosis of the tumour cells inhibits tumour growth (Xia et al., 2019). Drugs used to induce tumour cell death can produce anti-tumour immunity, resulting in tumour regression. It has been reported that when drugs are used to induce tumour pyroptosis it leads to anti-tumour immunity (Wang et al., 2020). The pyroptosis gene GSDMD may not only participate in regulating macrophage infiltration and polarisation but also influence the response to temozolomide in GBM (Liu et al., 2021). Research evidence has illustrated that pyroptosis-related gene signatures have a good prognostic value (Yang Z. et al., 2022; Zhang et al., 2022; Zheng et al., 2022). Nevertheless, research reports on pyroptosis-related lncRNA (PRLs) and its prognostic value in gliomas are scarce. This study sought to probe into the prognostic significance of PRLs, develop a classifier to foresee the survival outcome of patients with gliomas, and provide a clinical guideline and new insights into the concept of “personalised medicine” in glioma. We also intend to discover the most significant lncRNAs that regulate pyroptosis in gliomas to identify an effective therapeutic target for treating gliomas. Figure 1 illustrates the study’s flow chart.

## Materials and methods

### Collection of data on patients with gliomas

Data on transcriptomes, somatic mutations, and clinical characteristics of patients in the TCGA cohort were obtained from The Cancer Genome Atlas (TCGA). While data on transcriptomes and clinical parameters of patients in the CGGA_693 and CGGA_325 cohorts were extracted from the Chinese Glioma Genome Atlas (CGGA) (Zhao et al., 2021). Patients without data on the survival time and those who lived for less than 30 days after diagnosis were excluded from our study. Finally, we included data from 1576 glioma samples. Transcriptomic data of TCGA and CGGA_693 were analysed to identify the prognostic PRLs. In the subsequent development of the classifier, the TCGA dataset was chosen as the training set, while the CGGA_693 and CGGA_325 datasets were the validation datasets. Before the analysis, these transcriptomes of patients with gliomas were normalised to fragments per kilobase million (FPKM), except for the DESeq2 differential expression analysis. Table 1 summarises the clinical information of each dataset.

**TABLE 1:** Clinicopathological features of the patients in the TCGA, CGGA_693, and CGGA_325 cohorts.

Variables	TCGA (n = 611)	CGGA_693 (n = 656)	CGGA_325 (n = 309)
Age (Mean ± SD)	47.21 ± 15.10	43.43 ± 12.41	43.27 ± 11.93
Sex			
Male	343	374	194
Female	268	282	115
WHO grade			
II	219	172	97
III	235	248	73
IV	157	236	135
MGMTp status			
Methylated	431	304	151
Unmethylated	139	217	140
NA	41	135	18
1p/19q codeletion			
Codel	149	137	62
Non-codel	447	453	239
NA	15	66	8
IDH status			
Mutant	384	332	165
Wildtype	210	276	143
NA	17	48	1

### Discovery of prognostic pyroptosis-related lncRNAs

Previous studies provided us with 33 pyroptosis-related genes (Ye et al., 2021). The type of RNA aligns with the annotation of the Genome Reference Consortium Human Build 38 (GRCh38) to lncRNA would be considered. Then, the lncRNAs with Pearson’s r absolute value of >0.5 and *p*-value <0.001 were considered as PRLs. Lastly, PRLs linked to prognosis were determined *via* Cox regression analysis.

### Establishment and evaluation of the predictive potential of the PRL classifier

To select the significant lncRNA linked to the prognoses of patients with gliomas, the Boruta algorithm, an algorithm based on random forest, was used for feature selection (Kursa and Rudnicki, 2010). The lncRNA confirmed as the crucial feature for prognosis will be included in the subsequent study. To further evaluate the candidate lncRNA for the construction of the PRL classifier, we applied the least absolute shrinkage and selection operator (LASSO) algorithm and Cox regression. The classifier was referred to as the “risk score”. Below is the formula for risk score

$$risk\;score=\overset{n}{\underset{i=1}{\sum }}\left({Coef}_{i}*x_{i}\right)$$

Where ${Coef}_{i}$ denotes the coefficients, and $x_{i}$ denotes the FPKM value of 11 PRLs. The median value of the risk score was considered as a threshold value to distinguish between high- and low-risk score. We compared the survival times of the two groups utilising the “Survminer” package. The area under the curve (AUC) value calculated by the “timeROC” package was used to examine the predictability of the risk score and clinical factors linked to the prognosis of patients with gliomas, was derived utilising.

### Construction and verification of a nomogram

First, the risk variables of the clinicopathological parameters that might impact the prognosis of patients with glioma were determined by means of univariate Cox regression. Predicated on the prognostic factors, a nomogram was created using multivariable stepwise logistic Cox regression, and visualisation was done with the “rms” package in R. The prediction effectiveness of the nomogram was also evaluated by calculating the AUC values of each clinical feature using the “timeROC” package. The predictability of the nomogram was assessed using calibration curves and AUC values of each factor.

### Functional enrichment analysis

Differential expression analysis between the high and low risk score groups was performed using the “DESeq2” package. The “ClusterProfiler” package was applied to annotate the function of differentially expressed genes based on Gene Ontology (GO) and the Kyoto Encyclopaedia of Genes and Genomes (KEGG) pathways enrichment analysis. The enrichment score of each glioma sample was calculated through Gene Set Variation Analysis (GSVA). “Limma” package was applied to analyze the differentially activation of Reactome and KEGG pathways between high and low risk groups.

### Evaluation of the association between the PRL classifier and the glioma microenvironment and mutation profile

“CIBERSORT” was used to explore the infiltration levels of 22 distinct types of immune cells in the glioma samples (Newman et al., 2015). The stromal and immune scores, as well as the tumour purity of the glioma samples, were computed with the “ESTIMATE” package (Yoshihara et al., 2013). The tumour immune checkpoints were retrieved from past studies (Ping et al., 2021; Zheng et al., 2021). “Maftools” was used to visualise the mutation sites and calculate the tumour mutation burden (TMB) of each glioma sample.

### Prediction of drug sensitivity of glioma

The “oncoPredict” package was applied to predict the 50% inhibiting concentration (IC50) values of the glioma samples to various antineoplastic drugs in the Cancer Therapeutics Response Portal (CTRP) (Maeser et al., 2021). Then Spearman correlation analysis was implemented for IC50 values and risk score for identifying the sensitive and resistant drugs (with the |R | >0.5 and *p* < 0.05).

### Evaluation of the effectiveness of radiotherapy

Since there were only a few patients with GBM in the low risk group, we merged three datasets and grouped the patients based on their clinical features. Following this, we conducted a survival analysis to probe the efficacy of radiotherapy in each clinical subgroup.

### Prediction of the response to immune checkpoint blockade (ICB)

On the webpage http://tide.dfci.harvard.edu, an online computation was performed to determine the tumour immune dysfunction and exclusion (TIDE) score, which was applied to anticipate how gliomas respond to ICB. The cut-off value for predicting the patient’s response to ICB was set at 0.

### Acquisition of glioma samples and real-time quantitative polymerase chain reaction (RT-qPCR)

All glioma samples were obtained after the patients provided informed consent. The Research Ethics Committee of Zhujiang Hospital affiliated with Southern Medical University approved this research. In addition, 20 glioma tissue samples (nine LGGs and 11 GBMs) were obtained from patients who received surgical resection. TRIzol was utilised to obtain the total RNA from the samples. A reverse transcription kit (AG11718) was then employed to extract the genomic deoxyribonucleic acid (DNA) (gDNA) from RNA samples and synthesise the complementary DNA (cDNA). The amplification of cDNA was performed on QuantStudio 3&5 using SYBR green (AG11718). Gene expression was standardised using the housekeeping gene glyceraldehyde-3-phosphate dehydrogenase (GAPDH). The primers for cDNA amplification are depicted in Supplementary Table S1.

### Statistical analysis

Data analysis and visualisation were done with the help of the R software (4.0.4). The Fisher’s exact test and chi-square test were employed to explore if there was a significant variation in clinical characteristics between the groups with high and low risk score, respectively. When comparing non-normally distributed continuous variables, we adopted the Wilcoxon rank-sum along with Kruskal–Wallis tests. The significance level was established at a two-sided *p*-value of <0.05.

## Results

### Determination of the prognostic pyroptosis-related LncRNA

As per the annotation of GRCh38 to lncRNA, the TCGA dataset comprised 14,690 lncRNAs, and the CGGA_693 dataset comprised 3951 lncRNAs. Then 1074 and 782 prognostic PRLs were obtained from these two datasets using correlation analysis and univariate Cox regression analysis. These two datasets then shared 311 lncRNAs that were considered prognostic indicators. Finally, 11 PRLs, including *RP11-303E16.2*, *RP11-360L9.7*, *RP11-513M16.7*, *RP11-617F23.1*, *CTD-2521M24.6*, paired box interacting protein 1-antisense RNA 2 (*PAXIP1-AS2*), *RP11-428J1.5*, *RP11-158M2.3*, SET binding factor 2-antisense RNA 1 (*SBF2-AS1*), adenosine diphosphate ribosylation factor guanosine triphosphatase-activating protein-antisense RNA 1 (*AGAP2-AS1*), and *AP001469.9*, were selected to construct the classifier.

### Construction of the PRL classifier and validation of its predictability

**FIGURE 2:**
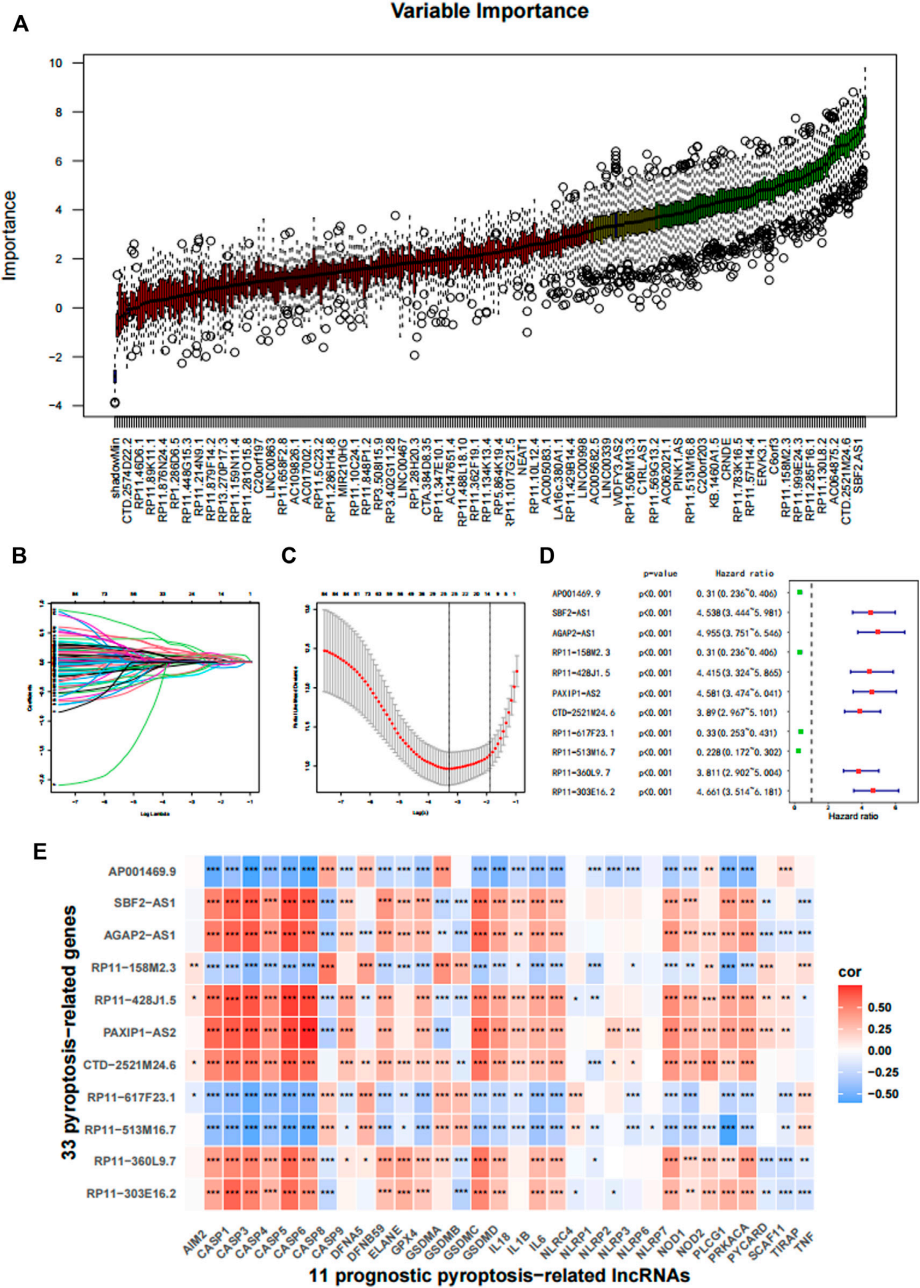
Construction of the prognostic pyroptosis-related lncRNA (PRL) classifier. **(A)** The Boruta algorithm was used to select important features. **(B,C)** The LASSO cox regression was conducted based on the minimum parameters. **(D)** The 11 PRLs were analyzed using univariate Cox regression. **(E)** The association of the 33 pyroptosis-related genes with 11 PRLs in TCGA cohort. *p < 0.05, **p < 0.01, and ***p < 0.001.

**FIGURE 3:**
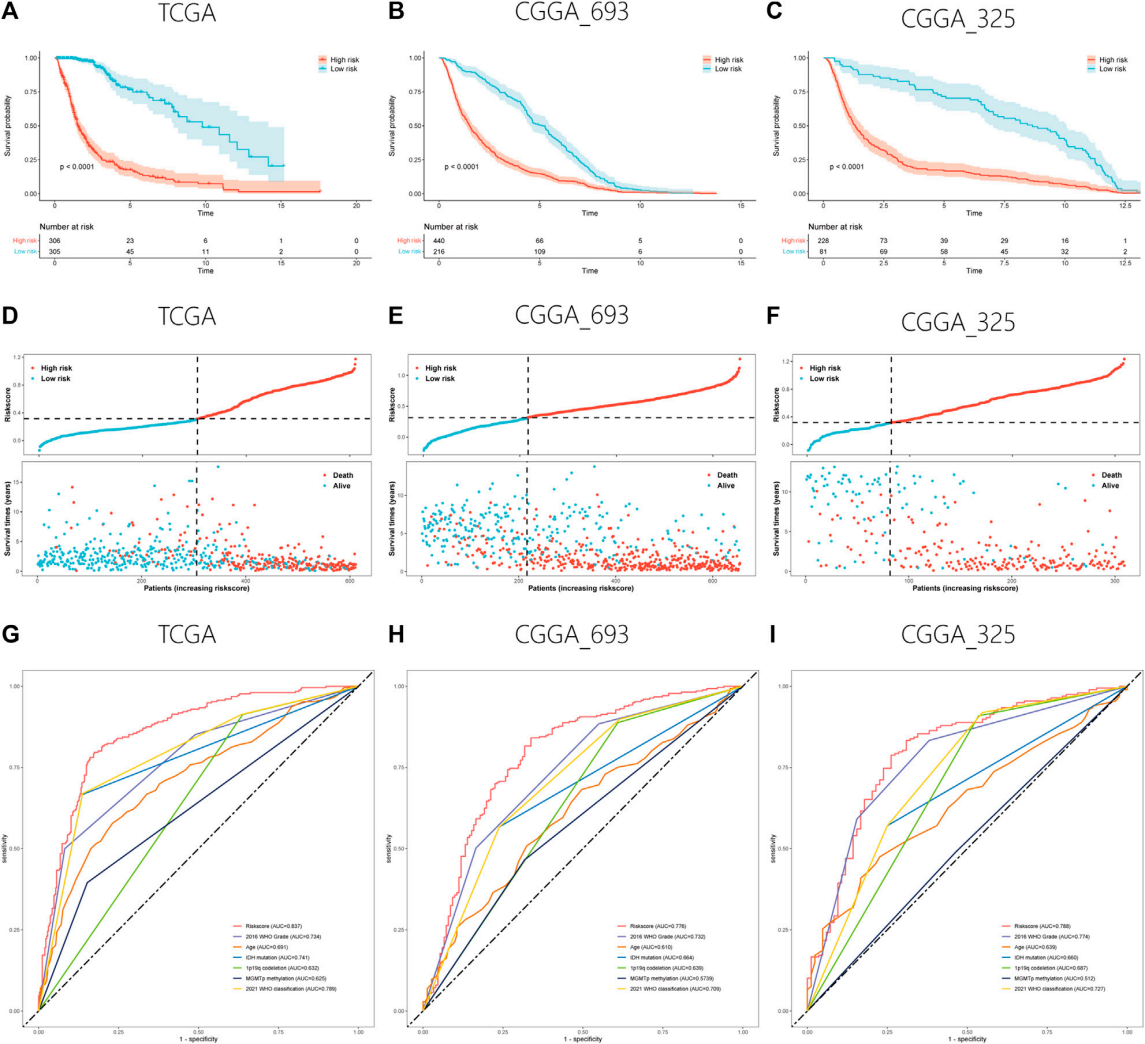
Validation of the PRL classifier. **(A–C)** The findings of KM curves for the three different cohorts in the TCGA, CGGA_693, and CGGA_325. **(D–F)** Representation of the TCGA, CGGA_693, and CGGA_325 cohorts’ risk score and survival status distribution plots. **(E–G)** Multiple ROC curves depicting the risk score as well as clinical variables for the TCGA, CGGA_693, and CGGA_325 cohorts.

After feature selection using the Boruta algorithm, 87 of the 311 prognostic PRLs were considered essential to anticipate the survival outcomes of patients with gliomas (Figure 2A). To simplify these signatures, the LASSO Cox algorithm and multivariate stepwise Cox regression were applied (Figures 2B,C). Figure 2D shows the univariate Cox regression coefficient of the 11 PRLs, suggesting that four of these PRLs are protective factors for glioma while the others are risk factors. Meanwhile, the correlations between 11 PRLs and 33 pyroptosis-related genes were presented in a heatmap (Figure 2E). When determining how to distinguish the high and low risk group, the median value was employed as the dividing line. Kaplan-Meier (KM) analysis highlighted that patients with a high risk score exhibited a dismal survival prognosis irrespective of the dataset (training or validation dataset) (Figures 3A–C). Similarly, it was observed that an increased risk score was associated with shorter survival time and a poor outcome (Figures 3D–F). The receiver operating characteristic (ROC) curve of the three datasets presented that risk score exhibited a larger AUC value than the clinicopathological parameters, indicating the relatively robust predictive power of PRL risk score (Figures 3G–I).

All the clinicopathological feature subgroups were compared for survival differences between the two classifications to explore whether the PRL classifier was suitable for all patients with gliomas. In most subgroups, patients with high risk score exhibited a dismal prognosis, except for a patient with GBM in the CGGA_325 cohort (Supplementary Figure S1).

### Association between risk score and the clinicopathological features of glioma

**FIGURE 4:**
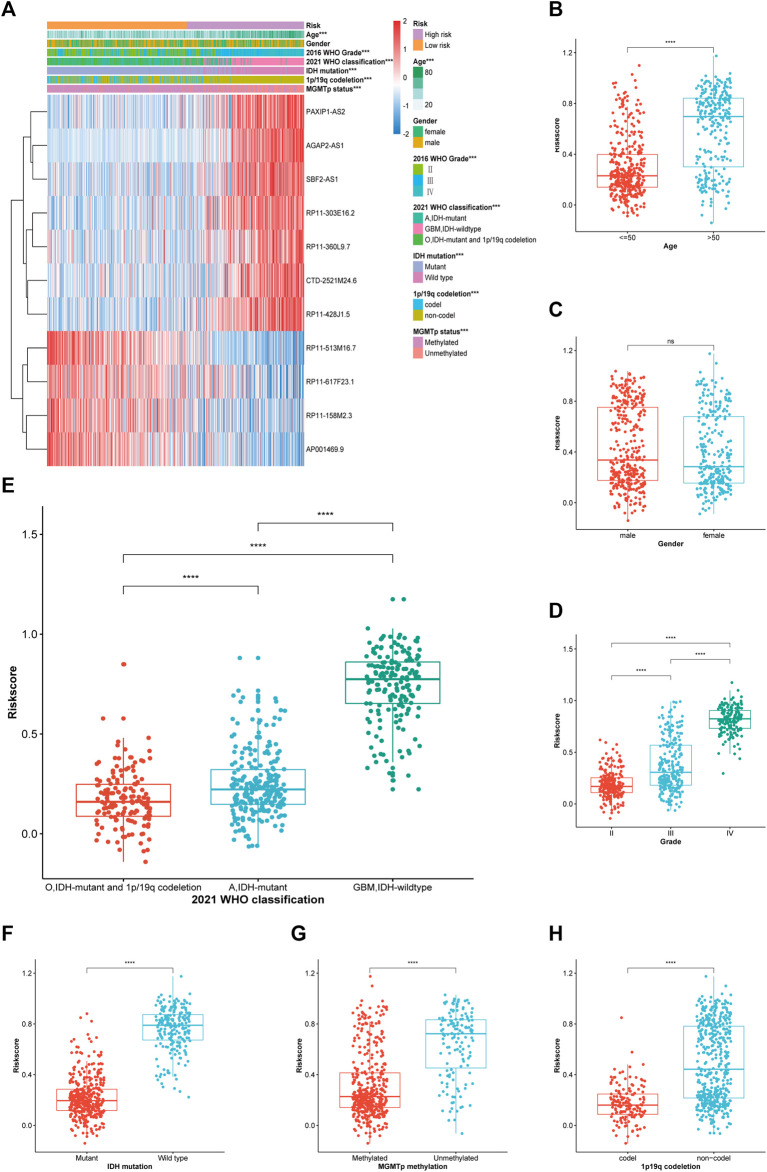
Correlation analysis between the PRL classifier and clinical-pathological parameters in TCGA cohort. **(A)** A heatmap illustrating the distribution of the clinical-pathological features and expression profiles of the 11 chosen PRLs in the high-and low-risk groups. **(B–H)** Various levels of risk scores in patients who had gliomas classified by: sex, age, WHO grade, 2021 WHO classification, IDH mutation status, 1p/19q codeletion, and MGMT promoter methylation. A, astrocytoma; O, oligodendroglioma; GBM, *p < 0.05, **p < 0.01, and ***p < 0.001. ns, no significance.

The proportion of the clinicopathological features suggesting a bad prognosis (isocitrate dehydrogenase [IDH] wild type, higher WHO grade, more malignant classification, etc.) was relatively high in the TCGA cohort (Figure 4A). However, both groups had similar proportions in terms of sex. Subsequently, the box plot revealed that elderly patients or those with a higher WHO grade or more malignant glioma had a higher risk score (Figures 4B–D). Patients with IDH wild type, chromosome 1p/19q codeletion, and O^6^-methylguanine-DNA methyl-transferase (MGMT) promoter unmethylation also had a higher risk score (Figures 4F–H). This finding was consistent in the CGGA_325 and CGGA_693 cohorts (Supplementary Figure S2), suggesting the generalisability of the risk score in glioma.

### Establishment of a nomogram

**FIGURE 5:**
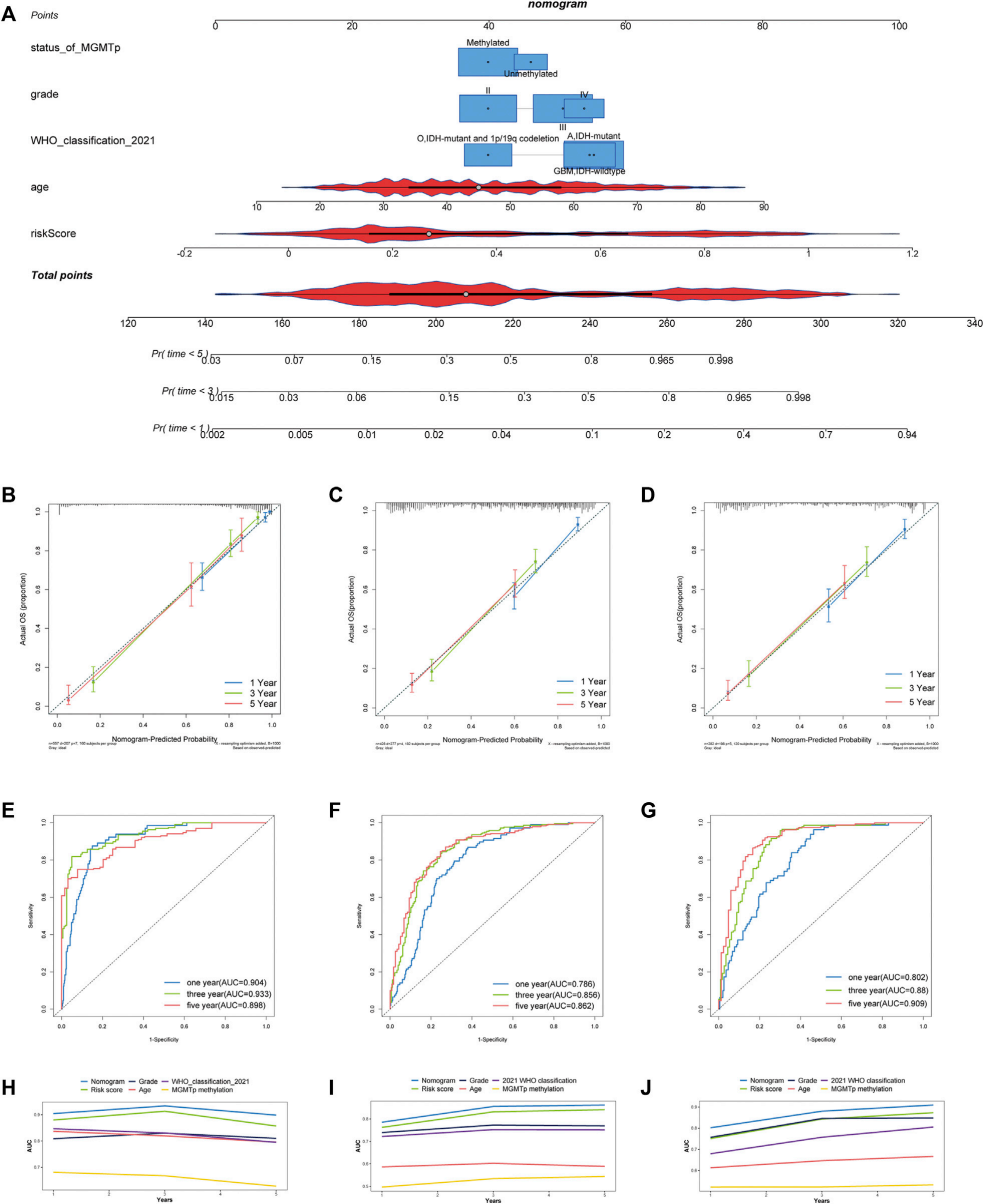
Development and assessment of a nomogram in TCGA cohort. **(A)** Nomogram based on the PRL risk score, age, WHO grade, MGMT promoter status and 2021 WHO classification. **(B–D)** Calibration curves that indicate the congruence between predicted and observed 1-, 3-, and 5-year overall survival (OS) in TCGA, CGGA_693 and CGGA_325 cohorts, respectively. **(E–G)** The receiver operating characteristic (ROC) curve analyses of the nomogram in predicting 1-, 3-, and 5-year OS in TCGA, CGGA_693 and CGGA_325 cohorts. **(H–J)** Line chart showing the AUC value of the nomogram, the risk score, grade, MGMT promoter status, age, and 2021 WHO grade in the TCGA, CGGA_693, and CGGA_325 cohorts.

Clinical and pathological features were combined to establish a nomogram for constructing a model that may efficiently anticipate the outcome of patients with gliomas. Univariate cox regression revealed risk score, patient age, 2016 WHO grade, 2021 WHO classification, MGMT promoter methylation, IDH mutation, and chromosome 1p/19q codeletion as significant prognostic factors (Table 2). A nomogram was then developed using risk score, 2016 WHO grade, patient age, the IDH mutation status, and chromosome 1p/19q codeletion status premised on the multivariate stepwise regression findings. The nomogram is presented in Figure 5A. The calibration curves revealed that the nomograms predicted survivor probabilities that were highly consistent with actual survivor probabilities intuitively (Figures 5B–D). As a result, the ROC plot showed excellent predictability for survival probabilities within 1, 3, and 5 years (Figures 5E–G). The increase in the predictive power of the nomogram compared with risk score and clinical features are intuitively clear from the line chart, irrespective of the cohort (TCGA, CGGA_693, or CGGA_325 cohorts) (Figures 5H–J).

**TABLE 2:** Univariate and multivariate Cox analyses in the TCGA, CGGA_693, and CGGA_325 cohorts.

Variables	Univariate analysis		Multivariate analysis	
	HR (95% CI)	*p*-value	HR (95% CI)	*p*-value
Age (Continuous)	1.065 (1.065–1.075)	<0.001	1.038 (1.025–1.51)	<0.001
Gender (Female vs. Male)	1.257 (0.978–1.616)	0.074	—	—
WHO grade				
II	1.000		—	—
III	3.377 (2.295–4.969)	<0.001	1.551 (0.999–2.409)	0.0508
IV	18.603 (12.563–27.549)	<0.001	1.758 (0.956–3.233)	0.0679
2021 WHO classification				
O, IDH mutant and 1p/19q codeletion	1.000			—
A, IDH mutant	1.647 (0.999–2.716)	0.0504	1.861 (1.103–3.140)	0.0200
GBM	12.566 (7.861–20.084)	<0.001	1.814 (0.957–3.438)	0.0679
Sample type (Primary vs. Recurrent)	1.438 (0.910–2.274)	0.120	—	—
IDH mutation (Mutant vs. wild type)	9.200 (6.962–12.16)	<0.001	1	—
1p/19q status (Codel vs. Non-codel)	4.474 (2.857–7.007)	<0.001	1	—
MGMTp status (Methylated vs. Unmethylated)	3.063 (2.323–4.039)	<0.001	1.285 (0.9283–1.779)	0.131
Riskscore (Continuous)	101.1 (60.93–167.8)	<0.001	21.169 (7.826–57.259)	<0.001

A, astrocytoma; O, oligodendroglioma; GBM, Glioblastoma.

### Functional enrichment analysis

**FIGURE 6:**
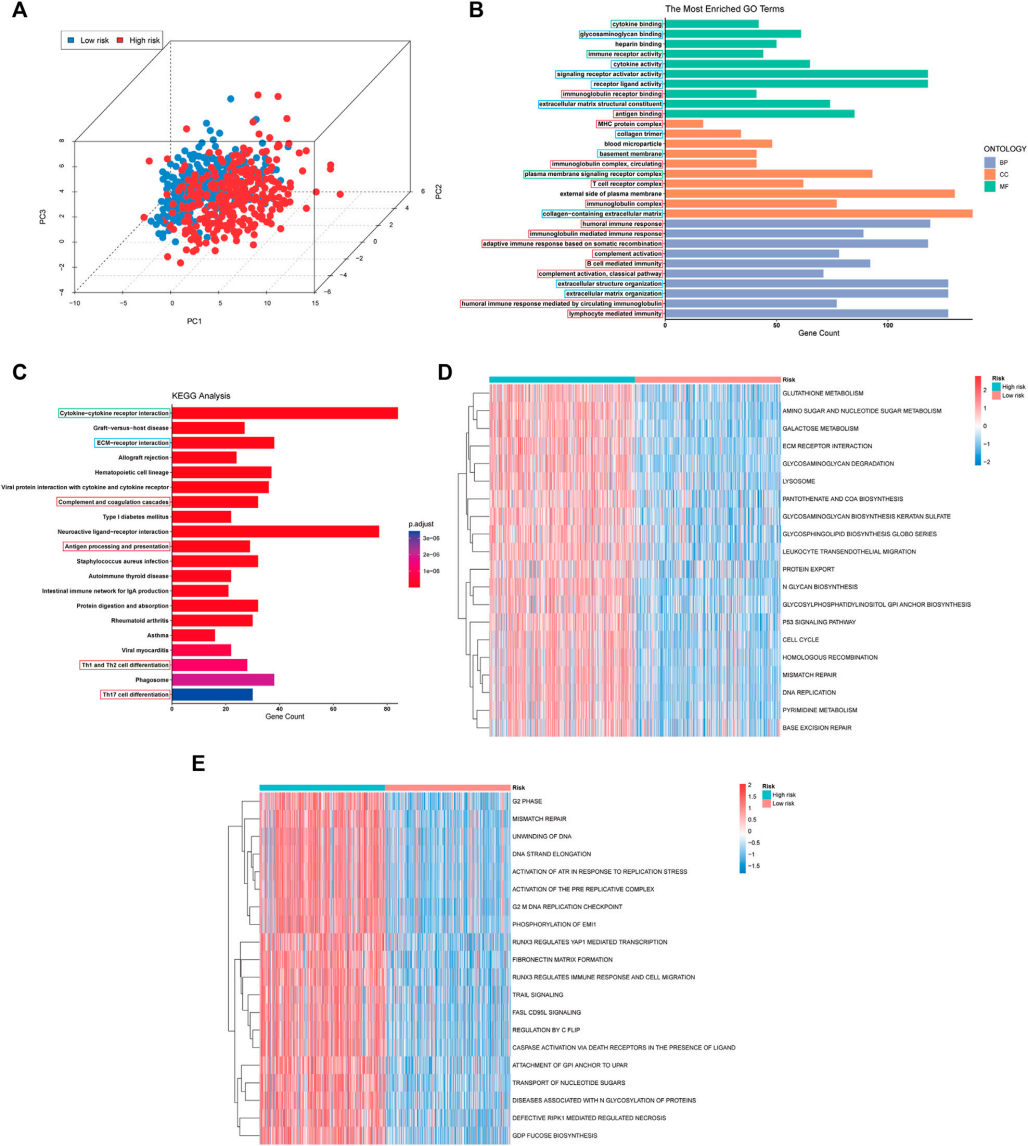
PCA plot and Functional enrichment analysis in TCGA cohort. **(A)** PCA showing the distribution differences between the high- and low-risk groups. **(B,C)** Results of GO and the KEGG analyses. The immune biological process, matrix-related pathway and intracellular communication pathways were boxed in red, blue, and green, respectively. **(D,E)** The top 20 differential activation pathways in KEGG and Reactome geneset between high and low-risk groups.

Firstly, the patient distribution in the high and low risk score groups was separated in the three-dimensional (3D) principal component analysis (PCA) plot, indicating, at least in part, the difference in the effect of pyroptosis or the degree of pyroptosis in glioma between the two groups (Figure 6A). The results of GO enrichment analysis showed that the 3912 differentially expressed genes (DEGs) were mostly enriched in immune-related pathways (immune receptor activity, immunoglobulin receptor binding, antigen binding, etc.), matrix-related pathways (extracellular matrix structural constituent, collagen trimer, collagen-containing extracellular matrix, etc.) and ligand-receptor interaction genset (cytokine binding, receptor ligand activity, signaling receptor activator activity, etc.) (Figure 6B). Among these immune-related pathway, humoral immunity-related pathways (antigen binding, immunoglobulin complex, circulating, humoral immune response, etc.) account for a large part. Similarly, the DEGs were also enriched in immune-related pathway (complement and coagulation cascades, antigen processing and presentation, Th1 and Th2 cell differentiation, etc.), matrix-related pathway (ECM-receptor interaction) and ligand-receptor interaction genset (cytokine-cytokine receptor interaction) based on the results of KEGG enrichment analysis (Figure 6C). Moreover, the results of KEGG GSVA showed the increased activation of cell proliferation related pathways (DNA replication, cell cycle, pyrimidine metabolism), DNA repaired related pathways (base excision repair, mismatch repair, homologous recombination) and metabolism related pathways (glutathione metabolism, amino sugar and nucleotide sugar metabolism, galactose metabolism) in the glioma with high risk score (Figure 6D). Similarly, the cell proliferation related pathways (G2 phase, DNA strand elongation, G2 M DNA replication checkpoint, etc.), DNA repaired related pathways (mismatch repair) and cell death related pathways (TRAIL signaling, FASL CD95L signaling, caspase activation *via* death receptors in the presence of ligand, etc.) were also upregulated in the high-risk group based on the results of Reactome GSVA (Figure 6E). Similar to KEGG and GO enrichment analysis, immune biological process genesets (RUNX3 regulates immune response and cell migration, leukocyte transendothelial migration) and matrix-related pathways (ECM receptor interaction, glycosaminoglycan degradation, glycosaminoglycan biosynthesis keratan sulfate) were also activated in the glioma with high-risk score. In brief, the results of enrichment analysis showed the differences in the immune biological process, matrix constitution, intercellular communication and cell metabolism between high and low risk groups.

### Metabolic heterogeneity and microenvironment of gliomas with different risk score

**FIGURE 7:**
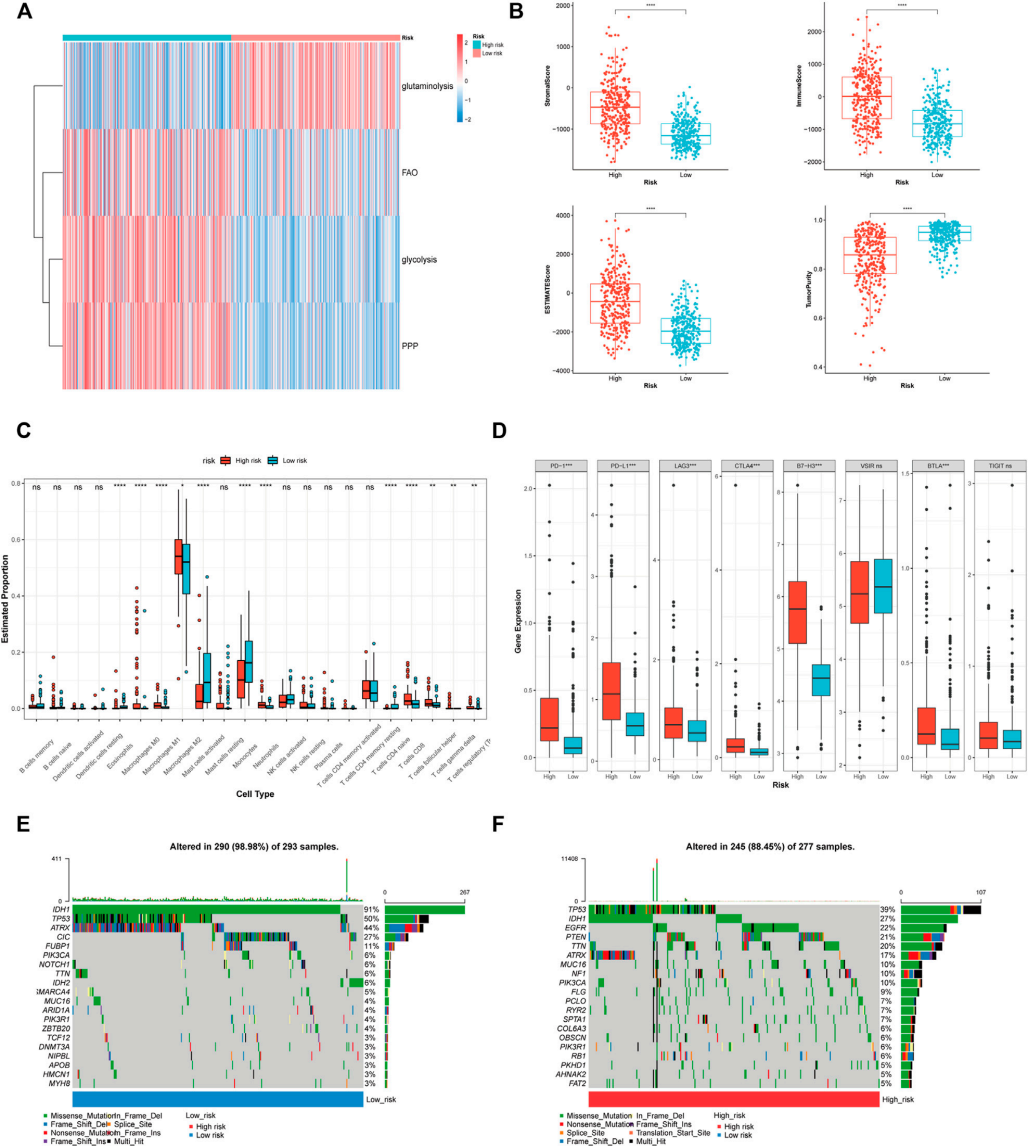
The correlation of PRL classifier with heterogeneous microenvironment in the TCGA cohorts. **(A)** GSVA showing the differences in the energy source between high and low risk group. **(B)** Comparison of the stromal, immune, and ESTIMATE scores, as well as the tumour purity between the high- and low-risk groups. **(C)** The abundance of 22 immune cells in the high- and low-risk groups. **(D)** The expression levels of the immune checkpoints in the high- and low-risk groups. **(E,F)** The top 20 genes mutation were visualized in the high- and low-risk groups. *p < 0.05, **p < 0.01, and ***p < 0.001. ns, no significance. FAO, fatty acid beta oxidation; PPP, pentose phosphate pathway.

Subsequently, we further explored the difference in metabolism, immunity, and matrix between high and low risk groups. Energy metabolism GSVA suggested that the energy metabolic pattern was depended on glycolysis, fatty acid oxidation and pentose phosphate pathway (Figure 7A). While glutaminolysis might be a crucial energy source for the glioma with low risk score. In terms of the differences in immunity and matrix, ESTIMATE analysis illustrated that patient having a high risk score exhibited significantly elevated stromal and immune scores and lower tumour purity (Figure 7B). Further investigation revealed that immune cells infiltrated gliomas with a high risk score (M2 macrophages, regulatory T cells, γδ T cells, and T follicular helper cells) and were mostly immunosuppressive (Figure 7C). However, certain anti-tumour immune cells (CD8^+^ T cells and M1 macrophages) were also found in higher proportions in the group with a high risk score. The immune cells enriched in the low-risk group were not special. The phenomenon of the CGGA_693 and CGGA_325 cohorts is largely consistent with that of the TCGA cohort (Supplementary Figure S3). Figure 7D shows that a high risk score was linked to an elevated immune checkpoint expression level, indicating an immunosuppressive microenvironment in the high risk group.

### The association between the PRL classifier and somatic mutation profile

The oncoplot showed that the mutations of tumour protein p53 (TP53), IDH, alpha-thalassemia/mental retardation, X-linked (ATRX), were prevalent in the high and low risk groups (Figures 7E,F). The frequency of the mutations that suggest a relatively good prognosis (IDH1, TP53, ATRX, and capicua) was lower in the high-risk group. Patients in the high-risk group exhibited an elevated frequency of mutations in epidermal growth factor receptor (EGFR) and phosphatase and tensin homolog (PTEN), indicating a poor prognosis. The difference in the somatic mutation between the two groups also reflected the accuracy of the PRL classifier in the prediction of glioma patients’ prognostic status.

### The association between the risk score and drug sensitivity

**FIGURE 8:**
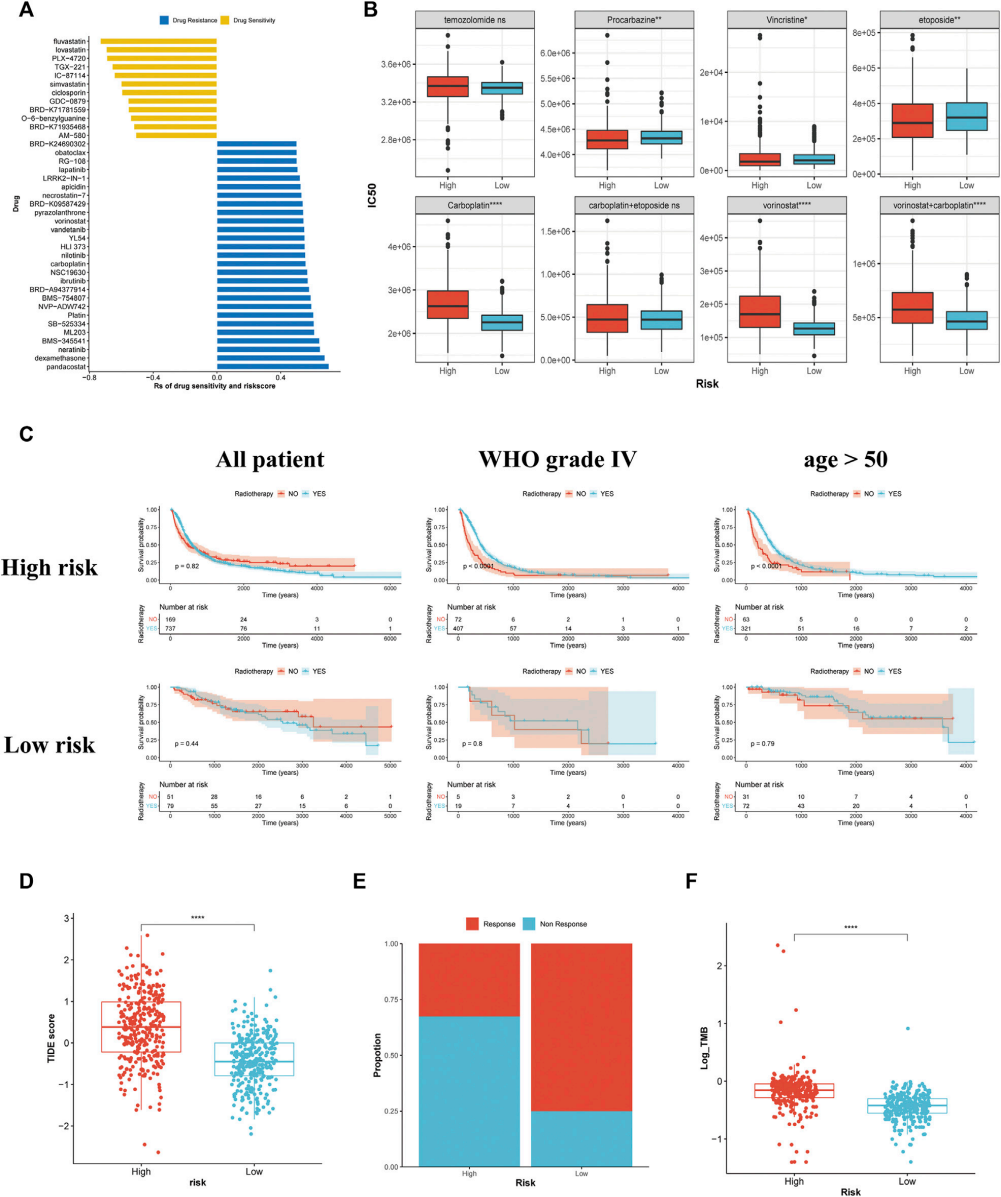
Association between the risk stratification and drug sensitivity, the effectiveness of radiotherapy, and the predictive response to immunotherapy. **(A)** The association of the risk scores with drug sensitivity evaluated using Spearman’s analysis. Each row represents a drug. The length of the row indicates the correlation, indicating that the risk score is related to drug resistance (Rs > 0) or drug sensitivity (Rs < 0). **(B)** The IC50 of the anti-glioma chemotherapeutic drugs in the high- and low-risk groups. **(C)** The KM curves for all patients, patients with glioma of WHO grade Ⅳ, and patients aged >50 years with or without radiotherapy in the high- and low-risk groups. **(D)** The TIDE scores of patients within the high- and low-risk groups. **(E)** The estimation of immunotherapy responsiveness in high- and low-risk groups. **(F)** The TMB levels of patients in the high- and low-risk groups.

The findings of Spearman’s correlation analysis revealed that the drug sensitivity of 40 antineoplastic agents was related to the risk score. Among these 40 drugs, 12 were relatively sensitive in the high-risk group while the others were relatively resistant (Figure 8). The 12 agents that were relatively sensitive in the high-risk group comprised 3-hydroxy-3-methylglutaryl-coenzyme A (HMG-CoA) reductase inhibitors (lovastatin and simvastatin), phosphatidylinositol-3-kinase (PI3K) pathway inhibitors (IC-87114 and TGX-221) and B-raf (BRAF) inhibitors (GDC-0879 and TGX-221). Most of the 28 agents relatively resistant in the high-risk group were platinum agents (carboplatin and platin), histone deacetylase inhibitors (apicidin, BRD-A94377914, pandacostat, BRD-K24690302, and vorinostat) and EGFR inhibitors (vandetanib, neratinib, and lapatinib). Further analysis among the six drugs recommended in the guidelines revealed that the sensitivities of temozolomide and carboplatin + etoposide in patients with gliomas patients did not vary considerably across the two groups (Figure 8). An elevated risk score was associated with greater sensitivity to procarbazine, vincristine, and etoposide but greater resistance to vorinostat, carboplatin, and carboplatin + vorinostat.

### The association between the PRL classifier and the effectiveness of radiotherapy

As demonstrated by the KM survival analysis, no remarkable variation in the survival outcome was observed among patients treated with radiotherapy and those who were not, regardless of their risk score. Patients aged >50 years and those with glioma of WHO grade 4 benefitted from radiotherapy, while patients with a low risk score did not benefit from radiotherapy (Figure 8C). Furthermore, the other subgroup of patients showed no significant difference in survival time (Supplementary Figure S4).

### Different classifications lead to different responses to ICB

Patients with a higher risk score had a higher TIDE score, indicating poor responsiveness to ICB (Figure 8D). The bar plot revealed that high-risk groups respond less frequently to ICB (Figure 8E). Subsequent analysis revealed that patients having a high risk score exhibited a higher TMB (Figure 8F). It was previously reported that gliomas with a high TMB showed poor responsiveness to immunotherapy (Samstein et al., 2019). Therefore, our TMB analysis might explain some of the variations across the two groups in terms of their responses to ICB.

### Expressions of the 11 prognostic PRLs in the glioma samples

**FIGURE 9:**
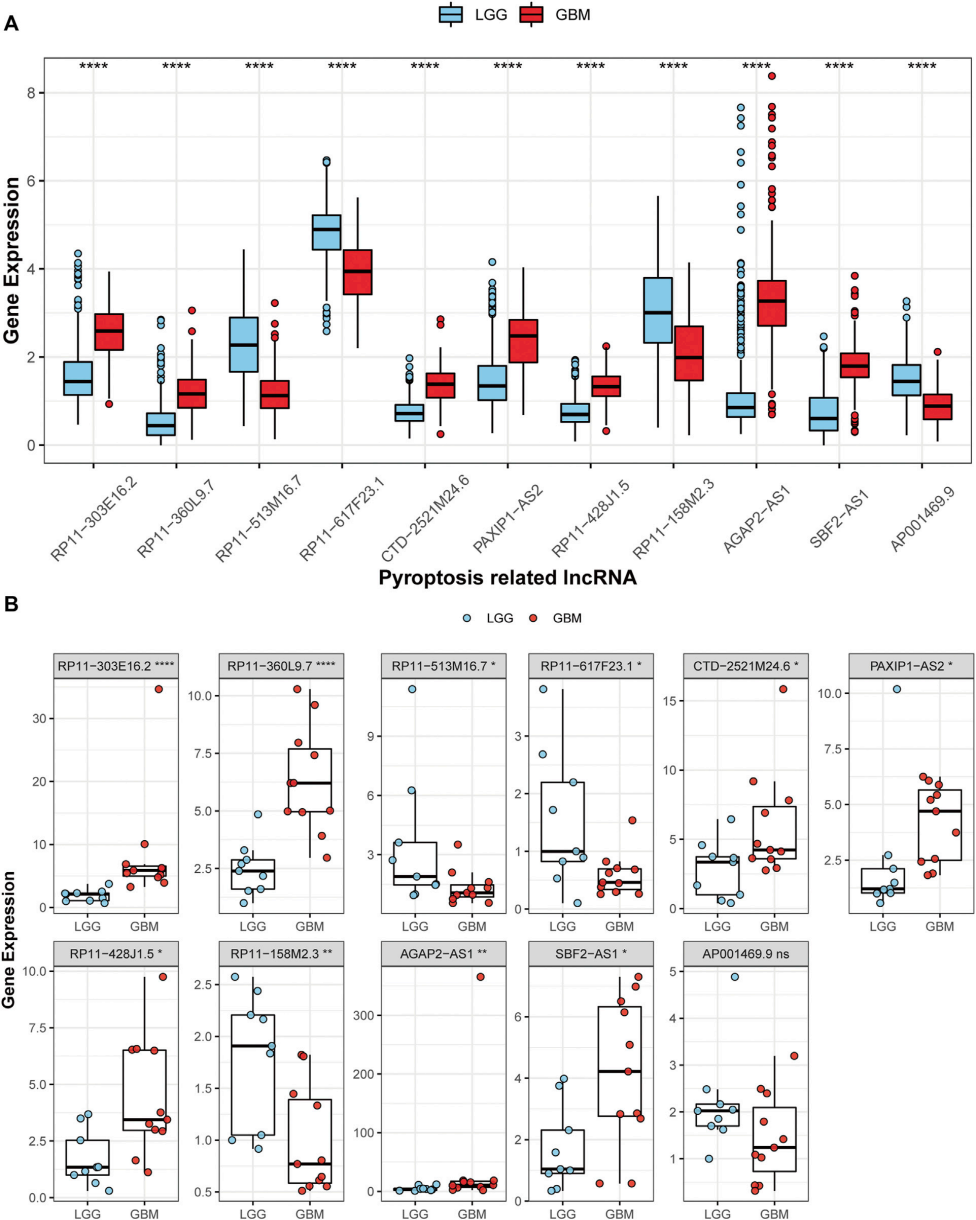
Validation of the 11 PRL expression levels. **(A)** The 11 PRL expression levels between LGGs and GBMs in TCGA cohort. **(B)** Expression analysis of the 11 PRLs in 9 LGG and 11 GBM samples. *p < 0.05, **p < 0.01, and ***p < 0.001. ns, no significance.

Among the 11 PRLs selected to construct the classifier, seven were up-regulated in GBM, while the others were down-regulated compared with LGG (Figure 9A). According to the results of the RT-qPCR, *CTD-2521M24.6*, *PAXIP1-AS2*, *RP11-303E16.2*, *RP11-360L9.7*, *RP11-428J1.5*, *AGAP2-AS1*, and *SBF2-AS1* were up-regulated in GBM, and *RP11-513M16.7*, *RP11-617F23.1*, and *RP11-158M2.3* expressions were significantly lower compared with LGG (Figure 9B). *AP001469.9* expression showed no difference between GBM and LGG. This result was in line with that of the TCGA cohort, highlighting the applicability of the PRL classifier.

## Discussion

Accumulating evidence suggests that tumour development is influenced by pyroptosis in a dual manner (Xia et al., 2019). Sustained release of inflammatory mediators by cells that undergo pyroptosis could promote epithelial-mesenchymal transition and build an immunosuppressive microenvironment, resulting in tumourigenesis. However, chemotherapy-induced pyroptosis could activate an immune response against tumours (Xia et al., 2019). Moreover, increasing studies have shown the feasibility and therapeutic potential of targeting distinct targets to trigger pyroptosis (Loveless et al., 2021). Nevertheless, pyroptosis in glioma has only been the subject of a limited amount of research efforts. The majority of research attention on pyroptosis was focused on the prognostication of pyroptosis-related genes. Few researchers investigated the prognostic value and underlying mechanism of lncRNA in pyroptosis regulation in glioma. Furthermore, growing evidence has demonstrated the crucial function of lncRNA in glioma progression regulation (Peng et al., 2018). Despite the same treatment, different effects are observed due to the heterogeneity of gliomas. Moreover, even patients with a similar pathological type of glioma have completely different prognoses. Therefore, it is important to establish a universal classifier for all glioma types as a prognosis-related predictor in patients with gliomas, thereby guiding clinical decision-making.

In our study, we first identified 311 prognostic PRLs on the basis of the CGGA_693 and TCGA cohorts. Among the prognostic PRLs, 11 PRLs (*RP11-303E16.2*, *RP11-360L9.7*, *RP11-513M16.7*, *RP11-617F23.1*, *CTD-2521M24.6*, *PAXIP1-AS2*, *RP11-428J1.5*, *RP11-158M2.3*, *AGAP2-AS1*, *SBF2-AS1*, and AP001469.9) were selected for construction of the PRL classifier. Among the 11 PRLs, four were protective factors, while the rest were risk factors. It has been reported that *PAXIP1-AS2* overexpression leads to a decrease in translesion DNA synthesis (TLS) by up-regulating the amount of RAD18 and DNA polymerase η (Swain et al., 2021). Furthermore, TLS inhibition increases the cell genomic incompleteness, resulting in tumourigenesis (Knobel and Marti, 2011). Therefore, *PAXIP1-AS2* might participate in tumourigenesis. Based on the RT-qPCR results, GBM exhibited higher *PAXIP1-AS2* expression than LGG. The higher expression of *PAXIP1-AS2* suggested that it could be a crucial factor for glioma development. The contribution of *PAXIP1-AS2* to tumourigenesis and progression needs further research. *AGAP2-AS1* could facilitate glioma growth by up-regulating hepatoma-derived growth factor (HDGF) (Zheng et al., 2019). In another study, *AGAP2-AS1* was considered a risky prognostic biomarker for the construction of a prognostic signature (Yu et al., 2021). This result illustrates the contribution of *AGAP2-AS1* to tumour progression, suggesting that *AGAP2-AS1* might be a target to suppress tumour growth. The lncSBF2-AS1-enriched exosomes induced chemotherapy resistance by remodelling the microenvironment (Zhang et al., 2019b). To stimulate angiogenesis, the nuclear factor of activated T cells 5 in GBM may upregulate SBF2-AS1 expression, which in turn could sponge miR-338-3p and elevate the HDGF expression level (Yu et al., 2017). This is consistent with our findings that *SBF2-AS1* is a risk factor in the PRL classifier. Although few studies have been reported on reaming PRLs, the RT-qPCR results of 11 PRLs in the glioma sample are broadly consistent with those of the TCGA dataset, illustrating that the differential expression of the 11 PRLs might be involved in glioma progression. However, we only explored the prognostic value of these 11 PRLs. Therefore, further research is warranted to explore the potential mechanisms of the 11 PRLs for pyroptosis regulation in gliomas.

The prognostic value and superiority of the PRL classifier were validated through KM survival analysis and a ROC plot. The survival rates of patients with a high risk score were quite dismal. The superior predictability of the risk score was presented using a ROC plot.

At the same time, the findings of the chi-square test illustrated that in the group with a high risk score, the proportion of clinical features suggesting a poor prognosis was higher. All results demonstrated the accuracy of the PRL classifier and the contribution of the 11 PRLs in influencing the prognosis of glioma. However, the prediction accuracy of the PRL classifier in glioblastoma subgroup needs to be further discussed. As the number of patients with GBM was low in the low risk group, the findings of the KM survival analysis might be inaccurate. Eventually, clinical characteristics linked to prognosis in glioma patients were used to generate a nomogram. Moreover, the predictability of the nomogram improved by the addition of a clinical covariate. Consequently, the nomogram may be a robust predictive tool for patients with gliomas.

Subsequent research revealed the underlying mechanism between the two groups. The 3D PCA plot revealed the difference in the degree of pyroptosis in glioma, suggesting the meaningful classification of the PRL classifier. And the results of GO and KEGG pathways enrichment analysis showed the differences in the immune biological process, matrix-related pathways, and receptor-ligand interaction between high and low risk group. This indicates that the 11 prognostic PRLs might affect the immune and stromal microenvironment, and intercellular communication of the glioma by regulating pyroptosis, leading to a difference in the prognosis of patients with gliomas. Antigen presentation, humoral immunity, and T cell differentiation are the main immune processes that differed between high and low risk groups. It has been reported that damage associated molecular patterns (DAMPs) released by pyroptosis cell could serve as immune adjuvant to enhance antigen presentation capacity of antigen-presenting cells and promote humoral immunity (Wang et al., 2022). However, the enhancement of humoral does not imply a better prognosis. A study showed that B cells located at the invasive margins could facilitate recurrence and progression (Zhang et al., 2019a). As for T cell differentiation, it has been shown that IL18, the cytokine released from pyroptosis cell, was not just involved in the differentiation of Th1 and Th2, but promoted the IL17 response in concert with IL23 (Esmailbeig and Ghaderi, 2017). And the anti-tumor effect of Th1, the pro-tumor effect of Th2 and the dual role of the Th17 in tumor might be the potential causes affecting the prognosis of the patients. While the relationship between pyroptosis and matrix remodelling has previously been reported. It has been reported that IL-1β can reduce the expression of the matrix components collagen type II and aggrecan in chondrocytes (Guo et al., 2021). In breast cancer, IL-1β has also been shown to up-regulate matrix metalloproteinase (MMP) 2, and MMP9, thereby promoting invasiveness and vasculogenic mimicry of tumour cells (Nisar et al., 2021). Research has shown that IL-18 promoted the invasive ability of HL-60 human myeloid leukaemia cells by up-regulating MMP9 expression (Zhang et al., 2004). And the cytokine released during pyroptosis and the interaction between cell and matrix might account for the differences in intercellular communication. While cell-cell communication in tumor microenvironment is related to tumor progression and metastasis (Fang et al., 2018). A more complex intracellular communication may create a more unstable tumor environment, leading to a completely different outcome of the patients. While the results of GSVA broadened our understanding about the different prognosis of patients in the high and low risk group. We found that the cell metabolism, cell death and proliferation related pathways and DNA repair related pathways were increased activation in the high-risk group. The metabolism of glutathione, amino sugar/nucleotide sugar and galactose was increased activate in the high-risk group. It has been reported that excess glutathione was correlated with tumor metastasis and progression (Fang et al., 2018). And the enhancement of amino sugar/nucleotide sugar metabolism would provide feedstock for the hexosamine pathway, leading to the glycosylation of lipids and proteins. For example, previous study found that the increased amino sugar/nucleotide sugar metabolism would increases complex N-Glycan structures and intracellular OGlcNAcylation, leading to tumor progression (Kim et al., 2020). Besides, a study suggested that the GBM could use galactose as energy sources (Sharpe et al., 2021). Therefore, the galactose could serve as an additional energy source for gliomas with high risk score in inadequate tumor perfusion environments, which may contribute to tumor progression. Thus, the differences in metabolism pathways might be responsible for the different survival outcomes between two groups. However, the underlying mechanism of metabolic reprogramming by the 11 PRL are still poorly understood. Another finding was that the cell death and proliferation related pathways were activated simultaneously in the high-risk group, suggesting that the gliomas might adapt and change the tumor microenvironment *via* this pattern. For example, the efferocytosis of the death cell could create an immunosuppressive microenvironment (Zhou et al., 2020). At the same time, the increased activation DNA repair related pathways in the glioma with high risk score may lead to a resistant to chemotherapy and radiotherapy (Bao et al., 2006). In summary, the 11 PRL might affect the immune biological process, matrix construction, metabolic reprogramming, and DNA preparation of the glioma, leading to a different prognosis of the patients in different groups.

The differences in the cell metabolism, immune biological process, and matrix constitution between two groups were further discussed. The glycolysis, fatty acid oxidation and pentose phosphate pathway were the main energy provider for glioma with high risk score. While the energy source of glioma with low risk score was depend on glutamine. The application of drugs targeting different metabolic pathways to patients in different groups according to their metabolic characteristics may offer new therapeutic strategies for glioma. Then the results of the ESTIMATE analysis revealed that the higher the inflammatory and stromal cell infiltrate and the lower the tumour purity in the glioma, the higher the risk score. CIBERSORT analysis revealed that gliomas with a high score comprised significantly more immunosuppressive immune cells, suggesting the presence of an immunosuppressive microenvironment in gliomas with high risk score. This is also illustrated by the differential expression of immune checkpoints between the two classifications. Collectively, these findings illustrate that the 11 PRLs might influence the formation of an immunosuppressive microenvironment and matrix remodelling of the glioma, leading to a dismal prognosis in patients with a high risk score. However, the infiltration level of some anti-tumour immune cells (M1 macrophages and CD8^+^ T cells) was relatively elevated in gliomas with high risk score. CD8^+^ T cells were found to be dysfunctional and failed to secrete sufficient tumour necrosis factor and interferon-γ to aid tumour regression (Philip and Schietinger, 2022). This explains why a higher proportion of CD8^+^ T cells is associated with poor survival outcomes. M1 macrophages demonstrated an anti-tumour effect in most studies (Hambardzumyan et al., 2016). However, the pro-tumour effect of M1 macrophages has also been reported. A study revealed that some inflammatory mediators secreted by M1 macrophages, namely, chemokine (C-C motif) ligand 5 and IL-6, were found to be tumour supportive, suggestive of a poor prognosis. Detailed discussion on this requires further research. This part of our study revealed the difference in the degree of pyroptosis in glioma and the immunosuppressive microenvironment and diverse metabolism of gliomas with high risk score. Moreover, a pyroptosis inducer exhibits an outstanding potential to activate a tumour cell-intrinsic immune response (Loveless et al., 2021), suggesting that a pyroptosis inducer might be suitable for treating patients with high risk score.

Currently, the primary treatment performed for gliomas is a comprehensive treatment based on a combination of surgery and chemotherapy, radiotherapy, and tumour treatment fields (TTF). Despite rapid advances in establishing an early diagnosis and treatment, nearly all gliomas become chemoresistant and metastatic. Our findings illustrate that patients with high risk score had a relatively enhanced sensitivity to HMG-CoA reductase inhibitors, PI3K pathway inhibitors, and BRAF inhibitors but were more resistant to platinum agents, histone deacetylase inhibitors, and EGFR inhibitors. Among the eight recommended regimens (Nabors et al., 2020), patients with higher risk score were found to be more sensitive to procarbazine, vincristine, and etoposide, while patients with lower risk score were more sensitive to vorinostat, carboplatin, and carboplatin + vorinostat. According to the current guidelines for glioma, radiotherapy is recommended for patients with high-grade and recurrent gliomas. However, it is unclear whether all patients with high-grade gliomas will benefit from it. Research has shown the crucial function of lncRNA nuclear enriched abundant transcript 1 in regulating the pyroptosis of HCT116 cells induced by ionising radiation (Su et al., 2021). This article suggests the critical function of pyroptosis in radiotherapy. Due to the difference in the sensitivity to pyroptosis, the effectiveness of radiotherapy might differ between the low- and high-risk groups. As per the findings of the KM survival analysis, patients aged >50 years or those with glioma of WHO grade Ⅳ benefitted from radiotherapy. This finding might guide clinical decision-making in terms of radiotherapy administration. Although immunotherapy is not the primary treatment for glioma, it has gained popularity. Hu5F9-G4, an anti-CD47 antibody, played a role in treating paediatric GBMs (Yang K. et al., 2022). The cytotoxic T-lymphocyte-associated antigen 4 antibody ipilimumab combined with nivolumab, plays a role in recurrent GBMs (Yang K. et al., 2022). However, the anti-programmed death 1 antibody pembrolizumab is ineffective in most gliomas, except for patients with special mismatch repair defects (Yang K. et al., 2022). From the studies above, we found that immunotherapy for glioma is developing. Nonetheless, immunotherapy may only be effective in specific patients. The TIDE scores revealed that patients having a higher risk score might not gain benefit from the ICB. Subsequently, the comparison of the TMB between the two groups verified the accuracy of the prediction. Therefore, other treatment regimens are required.

Indubitably, there are still significant limitations to our study. First, the medical history, tumour size, tumour location, and other factors associated with survival outcomes across the low- and high-risk groups were not matched. Second, the significance of the 11 PRLs in tumourigenesis cannot be explained because of the lack of non-tumour tissue. Further research involving larger samples is warranted to support the findings of our study. Third, the mechanism behind the involvement of the 11 PRLs in pyroptosis regulation in gliomas is not well known, necessitating additional research. The current studies are retrospective in nature, based on public databases and predictions. A prospective study to assess the clinical application of the PRL classifier would be more convincing.

## Conclusion

This study developed a PRL classifier and a nomogram as predictors for the survival outcome of patients with gliomas. Furthermore, the different immune landscapes between the two classifications helped us understand the underlying mechanisms of PRL in pyroptosis regulation and shaping the microenvironment of glioma. Simultaneously, the PRL classifier helped in clinical decision-making regarding radiotherapy, chemotherapy, and immunotherapy. We anticipate that these findings will help researchers and doctors in performing subsequent research and clinical work.

## Data Availability

The original contributions presented in the study are included in the article/Supplementary Materials, further inquiries can be directed to the corresponding authors.

## Supplementary material

SUPPLEMENTARY FIGURE S1 KM curves for the different subgroups of patients in TCGA **(A)**, CGGA_693 **(B)**, and CGGA_325 **(C)** datasets.

SUPPLEMENTARY FIGURE S2 Relationship between the risk score and glioma clinicopathological parameters in the CGGA_693 dataset **(A)** and CGGA_325 dataset **(B)**. A, astrocytoma; O, oligodendroglioma; GBM, glioblastoma. **p* < 0.05, ***p* < 0.01, and ****p* < 0.001. ns, no significance.

SUPPLEMENTARY FIGURE S3 Correlation between the PRL classifier and the microenvironment in CGGA_693 and CGGA_325 cohorts. **(A,D)** The infiltration degree of the 22 inflammatory cells between the two classifications. **(B,E)** The expression of the eight immune checkpoints between the two classifications. **(C,F)** The stromal, immune, and ESTIMATE scores, along with tumour purity between the two classifications. **p* < 0.05, ***p* < 0.01, and ****p* < 0.001. ns, no significance.

SUPPLEMENTARY FIGURE S4 KM survival curves for the different subgroups of patients with glioma in the high- and low-risk groups respectively.
