# Supplementary figures and images for: A prognostic pyroptosis-related LncRNA classifier associated with the immune landscape and therapy efficacy in glioma
Source: Front Genet. 2022 Oct 24;13:1026192. doi: 10.3389/fgene.2022.1026192 (PMC9637659; doi:10.3389/fgene.2022.1026192)

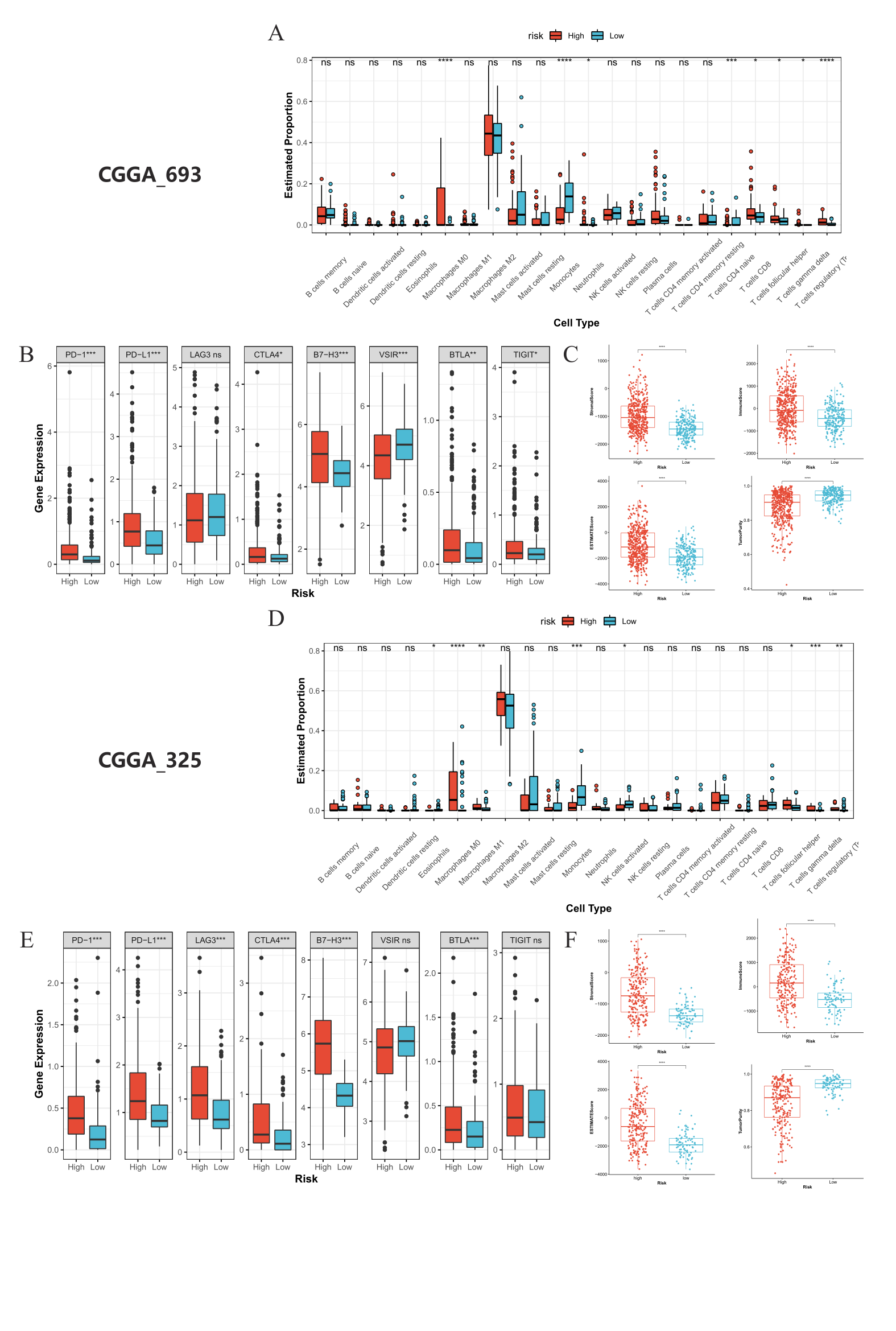

Supplement: Supplementary file 1 [file Image3.TIFF]

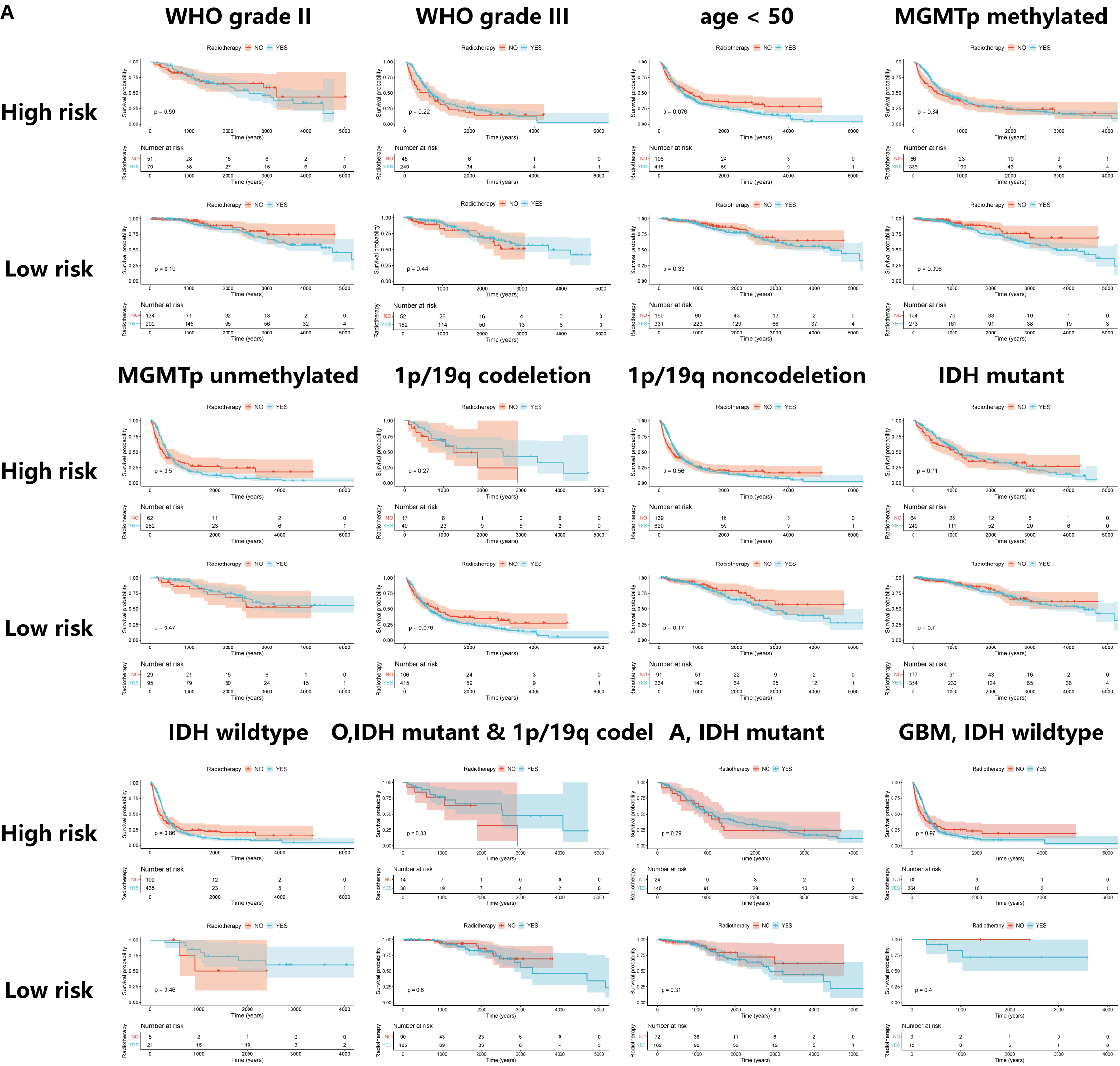

Supplement: Supplementary file 2 [file Image4.TIF]

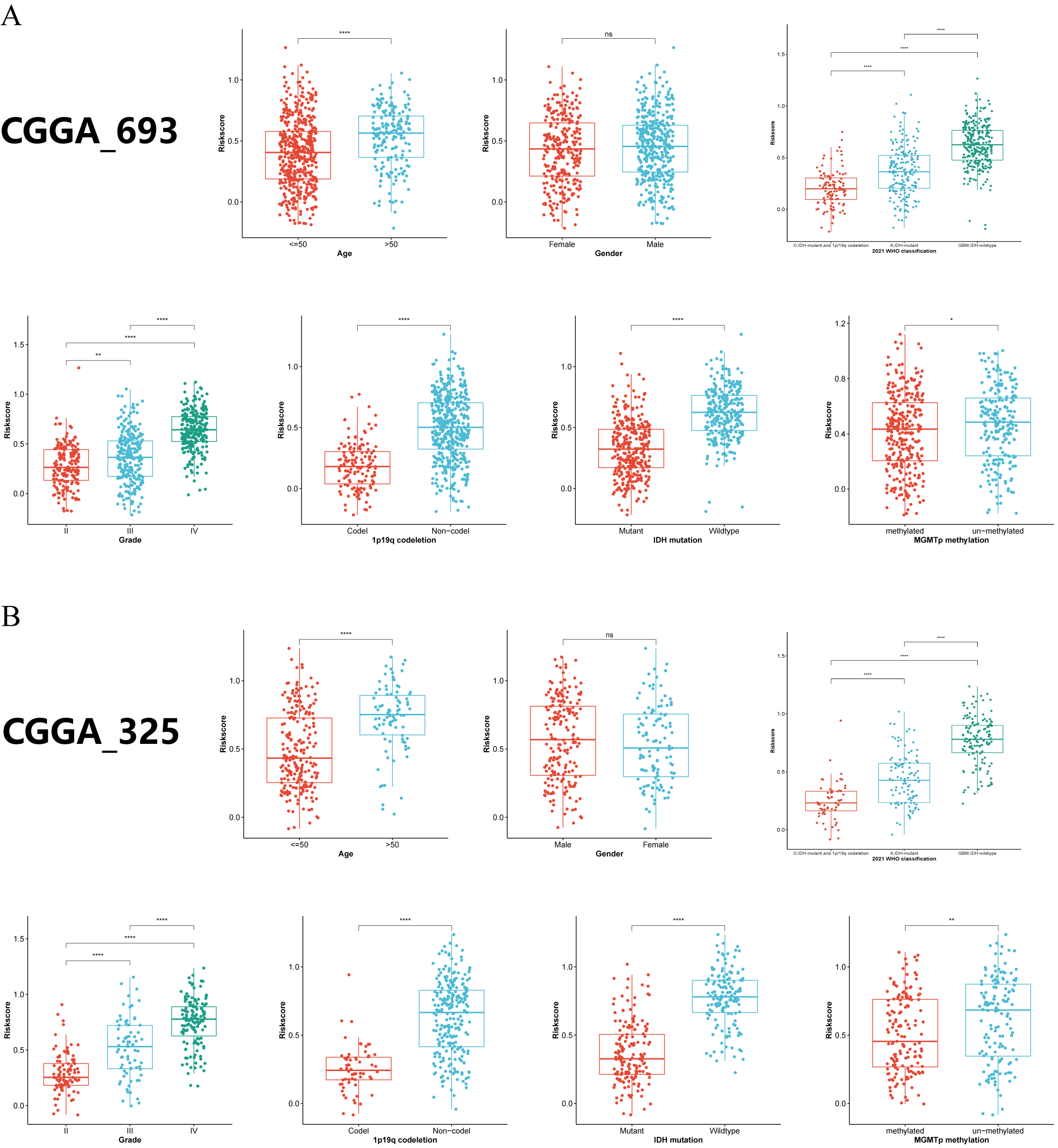

Supplement: Supplementary file 3 [file Image2.TIF]

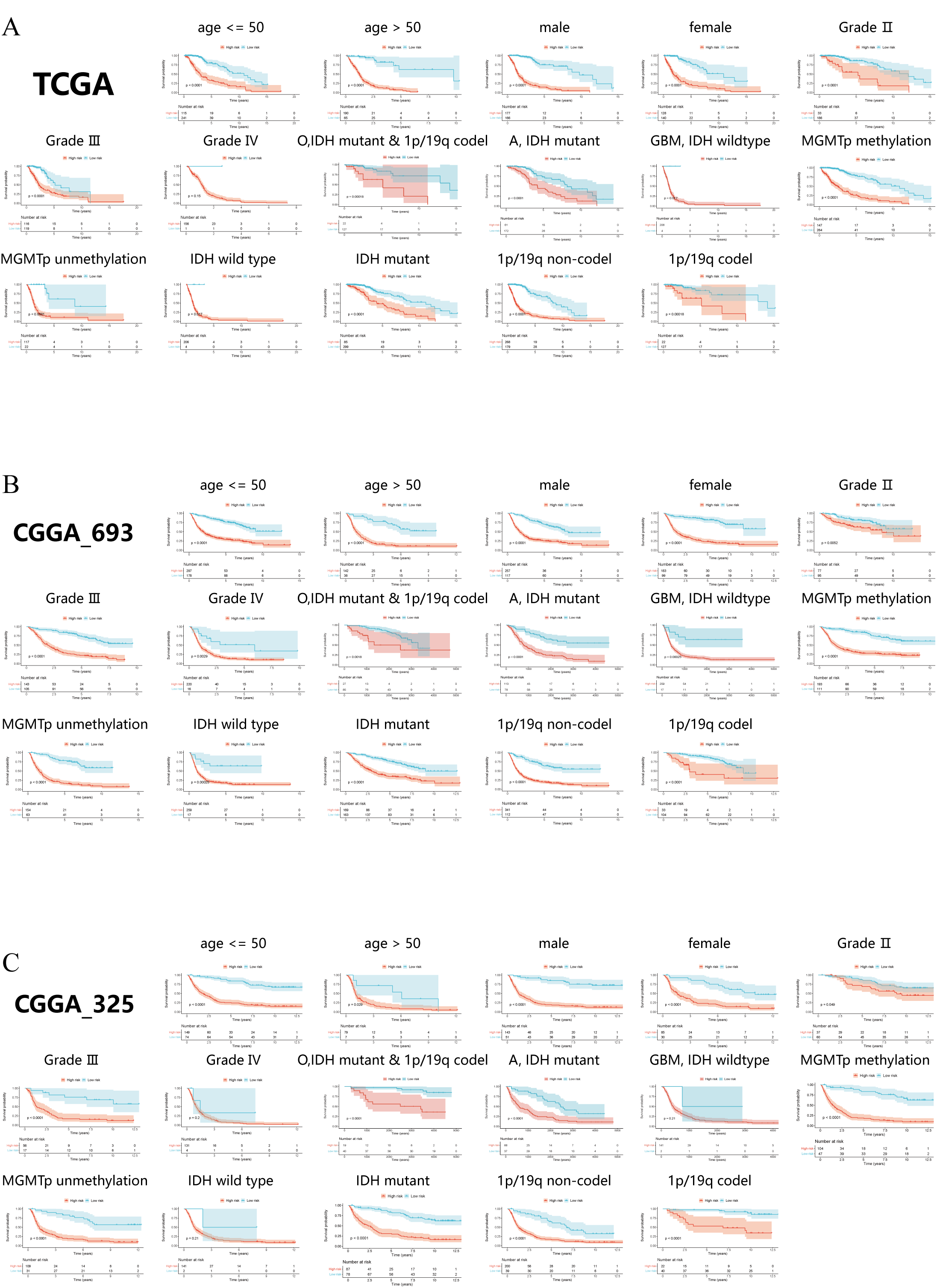

Supplement: Supplementary file 4 [file Image1.TIF]
