# Supplementary material for: A prognostic pyroptosis-related LncRNA classifier associated with the immune landscape and therapy efficacy in glioma
Source: Front Genet. 2022 Oct 24;13:1026192. doi: 10.3389/fgene.2022.1026192 (PMC9637659; doi:10.3389/fgene.2022.1026192)
Supplement: Supplementary file 5 [file DataSheet1.docx]

Supplementary Table S1 | The Primers sequence used in this study

| **Name** | **Forward-primer** | **Reverse-primer** |
| --- | --- | --- |
| RP11-303E16.2 | 5’-TTGAATTGGGCCAAATTGCGATGAC-3’ | 5’-TTGGTGCTGTTGAGGAGTTGTAGTG-3’ |
| RP11-360L9.7 | 5’-TGGAGGCATGAGGACGTAGAGC-3’ | 5’-TGAAACAGGGTGAGGTCCAGGATC-3’ |
| RP11-513M16.7 | 5’-GTGTCACATTTGGAGGAAGCTGTG-3’ | 5’-TGGAATAGAAACCTGCAGGAAGTTACA-3’ |
| RP11-617F23.1 | 5’-GCCAGGATTATAGGTGTGAGCCATC-3’ | 5’-ACAGGAAGGAGGGAGCCAACAG-3’ |
| CTD-2521M24.6 | 5’-AGGCAGCCACATGGACAACATTG-3’ | 5’-ATCAGCCTACTCCAGCGGTTCC-3’ |
| PAXIP1-AS2 | 5’-GTGAGCACTCCAGTCAGAACGAAC-3’ | 5’-CACGCACAGCAGTGGATCTCTAAC-3’ |
| RP11-428J1.5 | 5’-TGCATGGAGCATAGCGTCAC-3’ | 5’-CATGTTTAGGGCACCGCCTC-3’ |
| RP11-158M2.3 | 5’-CACACAGGAGACCACAGGCT-3’ | 5’-TCACCACCTGAGGGCATGAG-3’ |
| AGAP2-AS1 | 5’-CAGTCCTCCACTCCACCTCA-3’ | 5’-CCAGGCCATCGAGAGTCAGA-3’ |
| SBF2-AS1 | 5’-GAGGAAAGCCACGAGCACTG-3’ | 5’-CAGCCACAGGACATGCTGAA-3’ |
| AP001469.9 | 5’-GCTGGTCTCAAACTGGGCTCAAG-3’ | 5’-CACACAGGAGACCACAGGCT-3’ |
| GAPDH | 5’-AAAAGCATCACCCGGAGGAGAA-3’ | 5’- TCAAACTCCTGGGCTCAAGCAATC -3’ |

Supplementary Table S2 | The comparison of clinicopathological characteristics between the high-risk and low-risk groups in the TCGA cohort

| Clinicopathological characteristics | High-risk  ( n= 306) | Low-risk  ( n=305 ) | p-value |
| --- | --- | --- | --- |
| Age( Mean±SD ) | 53.60±14.99 | 40.80±12.25 | <0.001 |
| gender |  |  | 0.351 |
| Male | 128 | 140 |  |
| Female | 178 | 165 |  |
| WHO grade |  |  | <0.001 |
| II | 34 | 185 |  |
| III | 116 | 119 |  |
| IV | 156 | 1 |  |
| Histology |  |  | <0.001 |
| AA | 68 | 47 |  |
| A,NOS | 10 | 45 |  |
| GBM | 156 | 1 |  |
| AO | 30 | 86 |  |
| Mixed glioma | 25 | 45 |  |
| O,NOS | 17 | 81 |  |
| MGMTp status |  |  |  |
| Methylated | 148 | 283 | 151 |
| Unmethylated | 117 | 22 | 140 |
| 1p/19q codeletion |  |  | <0.001 |
| Codel | 23 | 126 |  |
| Non-codel | 268 | 179 |  |
| IDH status |  |  | <0.001 |
| mutant | 86 | 298 |  |
| wildtype | 206 | 4 |  |
